# Is Inner Speech the Basis of Auditory Verbal Hallucination in Schizophrenia?

**DOI:** 10.3389/fpsyt.2014.00075

**Published:** 2014-07-01

**Authors:** Raymond Cho, Wayne Wu

**Affiliations:** ^1^Department of Psychiatry and Behavioral Sciences, University of Texas, Houston, TX, USA; ^2^Center for the Neural Basis of Cognition, Carnegie Mellon University, Pittsburgh, PA, USA

**Keywords:** auditory hallucinations, schizophrenia, inner speech, self monitoring, spontaneous activation

We thank Moseley and Wilkinson (1) for their response to our article (2). Our aim was to contrast mechanisms of auditory verbal hallucination (AVH) to spur experimental work pitting models against each other, and we outlined experimental strategies to do so. While we favor a spontaneous activation model of AVH, different models might be needed to explain the panoply of AVH phenomenology (3). Here, we reconsider self-monitoring approaches that identify inner speech as the substrate of AVH.

We agree with Moseley and Wilkinson that inner speech is complex, in part because the term “inner speech” covers different phenomena. In a broad sense, it refers to a family of internal experiences of speech including (1) auditory imagination of one’s own or another’s speech and (2) internal articulation of one’s own thoughts in words [cf. (4); for potential distinctions in neural basis, see e.g., Ref. (5)]. To clarify our earlier discussion, it was the latter to which we referred with “inner speech,” what one could call inner speech in the narrow sense but which we will refer to as *internal articulation*. The challenge for inner speech theorists is to explain how one or more of these types of inner speech yields AVH.

This distinction between imagination and internal articulation bears on the study that Moseley and Wilkinson appeal to (6), which develops a questionnaire for probing the nature of inner speech. They claim that “the presence of other people’s voices is exactly the kind of quality reported in typical inner speech.” But is this typical? By far, the largest numbers of respondents (44%) claim that the presence of other people’s voices “certainly does not apply” to their inner speech. Indeed, the authors of the study only claim that “25.8% reported some other people in inner speech” and of these, only 7.8% claim that it “certainly applies to me” with the next strongest statement being that it “possibly applies to me” (8.7%). Furthermore, it is plausible that the questionnaire taps into the two different kinds of inner speech we have identified. The questionnaire can be divided into two sets of questions: those formulated with “thinking” and “talking” and those formulated with “hearing” when asking about other voices [Table 1 in Ref. (6)]. The first set might induce subjects to focus on internal articulation while the second induces them to focus on episodes of auditory imagination in which other voices might typically be experienced. If so, inner speech as auditory imagination might typically be of other voices, but it does not follow that internal articulation is typically of other voices. It is natural to think that when one internally articulates one’s own thoughts, inner speech is typically in one’s own voice. All this seems merely terminological, but it is not. The crucial point concerns not the labels we use but what the labels refer to, namely to what precise representations constitute the substrate of AVH. Given the ambiguity in “inner speech,” any theory invoking inner speech must specify the internal representation that serves as the substrate of AVH and explain how it yields AVH phenomenology. Only in this way can our hypotheses and questions be made clear and precise.

So, is the substrate of AVH internal articulation or auditory imagery (we set aside a third possibility, auditory recollection)? In objecting to self-monitoring theories, we focused on internal articulation, an experience typically in one’s own voice and lacking certain acoustical features common in AVH (7). While there is disagreement whether internal articulation is experienced as having volume [some deny this (8), some find 20% (9) of queried populations acknowledging this, and some as high as 90% (10)], it does seem that internal articulation is typically in one’s own voice where this rules out its exemplifying properties associated with experienced pitch and timbre distinctive of voices other than one’s own. Such properties are characteristic of AVH of other voices with specific genders, accents, and identities (11).

Any account that appeals to internal articulation as a substrate faces a challenge: because internal articulation typically lacks properties associated with the experience of pitch and timbre distinct from one’s own voice, self-monitoring accounts must explain the transformation of that substrate to AVH. “Transformation” here is used in a computational sense: there must be a process where the representations underlying internal articulation without certain acoustical features yield AVH with those features, namely those associated with a distinctive pitch and timbre tied to another voice. We do not claim that a transformation mechanism cannot be given, only that one must be provided. This has not been done.

Moseley and Wilkinson invoke work connecting AVH to subvocalization (12), which more naturally fits with internal articulation (“subvocalization” in the literature seems sometimes to refer to muscular activation without any produced speech, sometimes to sub-threshold speech). There has been little systematic follow-up work, however, and mixed results nailing down temporal correlation between muscle activity and AVH [for an overview, see Ref. (13)]. Moseley and Wilkinson note work shown by Bick and Kinsbourne (14) that in a group of schizophrenic patients, holding the mouth open during AVH abolished AVH in 72% of the patients. The putative mechanism, however, is puzzling. Readers might now try to generate inner speech while holding their mouths open. We find that we can do so, so the procedure does not seem to disrupt inner speech. It is not clear then how the result aids the inner speech model. A different explanation is that the patients at issue were in fact vocalizing, but at low volumes (12). If those actual sounds were the basis of AVH, then holding one’s mouth open could abolish AVH. Technically, however, these forms of “AVH” would not be hallucination of non-existent sounds but the misattribution of actual sounds. We doubt that all AVH involve actual vocalization and are thus mislabeled as hallucinations. Green and Kinsbourne (15) later failed to replicate the earlier result though some recent work has demonstrated lip muscle activity by EEG during AVH (16). The relevance of such activity to testing alternative theories, however, needs to be clarified.

There are other problems for appeals to internal articulation. Recently, McCarthy-Jones et al. (17) surveyed 199 individuals (65 female), 81% of whom were diagnosed with schizophrenia [the authors report that “the same 4-cluster structure (they identify) was found when the analyses were repeated, including only people with diagnosis of schizophrenia” p. 229 so we assume that the proportions apply to the schizophrenia subpopulation]. The data reveal forms of AVH that are difficult to explain by appeal to internal articulation as substrate: verbal gibberish AVH (21% of subjects), non-verbal auditory hallucination (music, animals, water, etc.; 32%), and multiple voices like a chorus (40%). These are experiences that one typically does not generate by internal articulation.

Accordingly, we offered a friendly suggestion to self-monitoring theorists (2): invoke auditory imagery as the substrate of AVH [see also Ref. (18)]. It is plausible that auditory imagery is like auditory experience in that both experiences represent acoustical properties such as intensity, pitch and timbre. Both appear to have a common basis in neural auditory representations (19). Thus, we think that between internal articulation and auditory imagery of other voices, the latter provides a *prima facie* more plausible substrate for AVH.

Having provided a friendly suggestion, we want to reiterate our main explanatory challenge to self-monitoring models: they are explanatorily incomplete at a crucial stage. The fundamental computation of most self-monitoring models draws on forward or predictive models from the motor control literature: the computation of the error between a predicted and actual signal. It is in this way that a system is said to monitor and track its outputs as self-produced. The problem is that computing error is far removed from the phenomenal properties characteristic of AVH. Alienness, otherness, loss of authorship/ownership or self-tags, and other descriptions characterizing AVH are phenomenological terms, but their connection to error signals is unclear. After all, error signals are computed in other domains having nothing to do with the phenomenology associated with AVH, say when in normal reaching, the motor system generates on-line correction of movement. Self-monitoring theorists need to close this gap in the explanation, and we are interested in clear answers that can be subject to empirical tests.

The spontaneous activation account provides straightforward explanations of some of these features. Consider the experience of otherness. Simply put, one experiences otherness because the substrate of AVH represents the voice of another. Moseley and Wilkinson object to this aspect of our model: “Taken to its extreme, [it] implies that any episode of inner speech that involves a voice other than one’s own would be experienced as ‘non-self,’ and hence experienced as similar to an AVH, a proposition that would clearly not find much support in empirical research.” Yet an experience of another’s voice by definition is experience of a non-self and in that way is qualitatively identical to AVH in respect of what is experienced: an other. Trivially, this “other” aspect of AVH is shared with auditory-based experiences of non-self voices whether in normal hearing, imagination, dreams, or memory. Each represents the voice of another. “Otherness” (non-self) as characterizing what is experienced in AVH is not mysterious on the spontaneous activation account [on pitfalls regarding talk of otherness; see Ref. (20) pp. 99–100]. While otherness is often distinctive of AVH, it is not sufficient to render AVH the mental disturbance that it is. Rather, it is also the specificity of content, acoustical properties, repetition and spontaneity of AVH episodes that exacerbate the negative impact of the symptom.

Moseley and Wilkinson also identify “the non-self-generated, alien quality associated with AVHs” as something to explain and claim that the spontaneous activity account cannot explain it. In respect of “non-self-generated,” the spontaneous account appeals to the spontaneity of AVH episodes that, like thoughts or tunes that pop into one’s head, have the phenomenology of not being self-generated. Again, this account demystifies one aspect of AVH phenomenology. The alien quality of AVH is more elusive though it is often invoked [e.g., Ref. (4); see Ref. (20), p. 89 for more references]. Like “inner speech,” “alienness” is hard to pin down. Until it is clear what it means, it is unclear what one should explain. This is why we have emphasized the importance of careful analysis, which is obligatory in describing complicated phenomenology. Perhaps “alienness” is a general expression of what is abnormal in AVH, but then the next step is to be clear what those abnormalities are and then to assess each model’s ability to explain them. “Alienness” is a too vague phenomenal descriptor, and until we better understand what it refers to, it would be better to not use it as an explanatory constraint in assessing theories. The first step, then, is to be clear what alien phenomenology is beyond it signaling something abnormal.

Moseley and Wilkinson suggest that our model does worse than self-monitoring models in explaining the specificity of the voice in AVH, but we disagree. Indeed, self-monitoring models have potentially two forms of specificity to explain: the specific failure of self-monitoring across types of inner speech (e.g., internal articulation versus imagination) and within each type, the specific failure of self monitoring for certain voices or sounds (e.g., auditory imagination of Barack Obama’s voice that yields AVH but not imagination of George Bush’s voice). On the spontaneous activation account, there will be corresponding overactivation of relevant auditory representations (increases in gamma synchrony could derive from the inappropriate activation of the *specific* neuronal assemblies that support such representations). All theories have to deal with the puzzling specificities associated with AVH (voices more than non-voices, auditory more than visual hallucinations, etc.). The spontaneous activity account does not seem worse on this point.

Finally, our aim was to motivate refinements of the issues by analyzing some of the key terms, questions, and mechanisms in the investigation of AVH. We agree with Moseley and Wilkinson that more work needs to be done on concepts and mechanisms.

## Conflict of Interest Statement

The authors declare that the research was conducted in the absence of any commercial or financial relationships that could be construed as a potential conflict of interest.
